# Supplementary material for: Patient preference and acceptability of self-sampling for cervical screening in colposcopy clinic attenders: A cross-sectional semi-structured survey
Source: PLOS Glob Public Health. 2024 May 23;4(5):e0003186. doi: 10.1371/journal.pgph.0003186 (PMC11115198; doi:10.1371/journal.pgph.0003186)
Supplement: S1 Questionnaire — (DOCX) [file pgph.0003186.s001.docx]

## S1: Questionnaire

**Low Vaginal Self-Sampling Acceptability Questionnaire St George’s Hospital Foundation NHS Trust**

1. Overall, how was your experience on collecting your own low vaginal self-sample?

- 1 = Extremely awful
- 2 = Awful
- 3 = Neutral
- 4 = Good
- 5 = Extremely good

1. How easy was it to collect the low vaginal self-sample?

- 1 = Extremely hard
- 2 = Hard
- 3 = Neutral
- 4 = Easy
- 5 =Extremely easy

1. How convenient was it to collect the low vaginal self-sample (in terms of privacy)?

- 1 = Not convenient at all
- 2 = Not convenient
- 3 = Neutral
- 4 = Convenient
- 5 = Extremely convenient

1. How embarrassed were you to collect this low vaginal self- sample?

- 1 = Extremely embarrassed
- 2 = Embarrassed
- 3 = Neutral
- 4 = Not embarrassed
- 5 = Not embarrassed at all

1. How much discomfort or pain did you experience while collecting this self-sample

- 1 = Severe discomfort/ pain
- 2 = Some discomfort/pain
- 3 = Neutral
- 4 = No discomfort/pain
- 5 = No discomfort/pain at all

1. How confident are you that you collected this self-sample correctly?

- 1 = Not confident at all
- 2 = Not confident
- 3 = Neutral
- 4 = Confident
- 5 = Very confident

1. Which method do you prefer the MOST for cervical screening?

- Self – sampling vaginal swab for HPV testing
- Physician – collected vaginal swab for HPV testing
- Pap smear – physician conducting a speculum examination
- No preference

1. Would you be willing to do the HPV self- sampling test again?

- Yes – please proceed to question 9
- No – please proceed to question 10

1. Why are you willing to do the HPV self-sampling test again? (Please select all that apply)

- The test is simple to do
- The test is quick
- I am confident that I can take own sample accurately
- I feel less embarrassed taking own sample
- I feel more comfortable taking own sample
- Taking sample with the swab was not painful

1. Why do you refuse to do the HPV self-sampling test again (Please select all that apply)?

- The test is not easy
- I am afraid I might hurt myself
- I am not confident to take own sample accurately
- I am not comfortable taking own sample
- Taking own sample with the swab was painful
- I prefer a healthcare professional to collect the sample

1. How did you find the Patient Information Sheet, PIS (Please select all that apply)

- 1 = It was very hard to understand
- 2 = It was hard to understand
- 3 = Neutral
- 4 = It was easy to understand
- 5 = It was very easy to understand

1. What can we do to improve your experience? Please add your comments.

____________________________________________________________________________________________________________________________________________________________________________________________________________________________________________________________________________________
