# Supplementary material for: Patient preference and acceptability of self-sampling for cervical screening in colposcopy clinic attenders: A cross-sectional semi-structured survey
Source: PLOS Glob Public Health. 2024 May 23;4(5):e0003186. doi: 10.1371/journal.pgph.0003186 (PMC11115198; doi:10.1371/journal.pgph.0003186)
Supplement: Attachment — Submitted filename: UKHSA PGPH-D-23-02495_reviewer comments.pdf [file pgph.0003186.s003.pdf]

PLOS Global Public Health

Patient preference and acceptability of self-sampling for cervical screening in colposcopy clinic attenders: a cross-sectional semi-structured survey

--Manuscript Draft--

**Manuscript Number:**	PGPH-D-23-02495
**Article Type:**	Research Article
**Full Title:**	Patient preference and acceptability of self-sampling for cervical screening in colposcopy clinic attenders: a cross-sectional semi-structured survey
**Short Title:**	Survey of self-sampling acceptability for cervical screening in colposcopy clinic
**Corresponding Author:**	Sophie Webb, MD (Res), BMBS<br>St George's University of London<br>London, UNITED KINGDOM
**Order of Authors:**	Sophie Webb, MD (Res), BMBS
	Nafeesa Mat Ali
	Amy Sawyer
	David J Clark
	Megan A Brown
	Yolanda Augustin
	Yin Ling Woo
	Su Pei Khoo
	Sally Hargreaves
	Henry M Staines
	Sanjeev Krishna
	Kevin Hayes
**Keywords:**	self-sampling; HPV; Cervical screening; acceptability; questionnaire
**Abstract:**	Low vaginal self-sampling has been pioneered as an important development to improve uptake of cervical screening globally. Limited research is available in specific patient groups in the UK exploring views around self-sampling to detect high-risk human papillomavirus (hrHPV) DNA. Therefore, we explored patient views to support development and evaluation of a novel point-of-care self-sampling cervical cancer screening device, by undertaking a cross-sectional semi-structured questionnaire survey to explore preferences, acceptability, barriers and facilitators around self-sampling.<br>Patients attending a colposcopy clinic (hrHPV negative and positive patients), 25-64 years old, were invited to participate after their appointment, having carried out a low vaginal self-sample. Participants self-completed an anonymous 12-point questionnaire. Quantitative data were analysed in MS Excel and Graphpad Prism whilst qualitative data were analysed using Nvivo.<br>We recruited 274 patients with a questionnaire response rate of 76%. Acceptability of self-sampling for screening was high (95%, n=187/197; Cronbachs-α =0.778). Participants did not report a strong single preference for future screening method, with 37% (n=74/198) preferring self-sampling, 19% (n=37/198) preferring healthcare professional-collected vaginal swab; 9% (n=17/198) a cervical sample with healthcare professional speculum examination, and 35% (n=70/198) had no preference. Key motivators for self-sampling were: Test simplicity (90%, n=170/190), speed (81%, n=153/190) and less pain (65%, n=123/190). Barriers included lack of confidence taking the sample (53%, n=10/19), resulting in preference for a healthcare professional to take the sample (47%, n=9/19).<br>Whilst self-sampling showed high acceptability, the lack of strong preference for screening method may reflect that respondents attending colposcopy are already

Powered by Editorial Manager® and ProduXion Manager® from Aries Systems Corporation

	engaged with cervical screening and have a differing perception of cervical cancer risk. This group appear less likely to 'switch' to hrHPV self-sampling, and it may be better targeted within primary and community care settings, focusing on under-screened populations. Any shift in this paradigm in the UK requires comprehensive education and support for patients and providers.
**Additional Information:**	
**Question**	**Response**
**Financial Disclosure**<br><br>Enter a financial disclosure statement that describes the sources of funding for the work included in this submission and the role the funder(s) played. This includes grants and any commercial funding of the work or authors.<br><br>This statement will be typeset if the manuscript is accepted for publication.<br><br>*Please review the submission guidelines and the instructions link below for detailed requirements and guidance.*	This work was support by the St. George's Hospital Charity.<br><br>HMS is supported by the Wellcome Trust Institutional Strategic Support Fund (204809/Z/16/Z) awarded to St George's University of London. www.wellcome.org<br><br>SH is funded by the National Institute for Health and Care Research (NIHR300072 and NIHR134801) www.nihr.ac.uk, the Academy of Medical Sciences - www.acmedsci.ac.uk (SBF005\1111), the La Caixa Foundation (La Caixa LCF/PR/SP21/52930003) - https://lacaixafoundation.org, Research England - www.ukri.org/councils/research-england, and WHO - www.who.int.<br><br>The funders had no role in study design, data collection and analysis, decision to publish, or preparation of the manuscript.
**Competing Interests**<br><br>On behalf of all authors, disclose any competing interests that could be perceived to bias this work.<br><br>This statement will be typeset if the manuscript is accepted for publication.<br><br>*Please review the instructions link below and PLOS Global Public Health's competing interests policy to determine what information must be disclosed at submission.*	SK and HMS are advisors to and shareholders in QuantuMDx, a molecular nucleic acid test-based diagnostic company. SK, YA and HMS are advisors to Global Access Diagnostics, a developer of rapid diagnostic tests, and SK is a member of the Scientific Advisory Committee for the Foundation for Innovative New Diagnostics (FIND), a not-for-profit organisation that produces global guidance on affordable diagnostics. These competing interests will not alter adherence to PLOS Global Public Health policies on sharing data and materials.
**Data Availability**<br><br>Before publication, Authors are required to make fully available and without restriction all data underlying their findings. Please see our PLOS Data	Available on St. George's University of London data repository: https://doi.org/10.24376/rd.sgul.24716424.v1

Policy page for detailed information on this policy.<br><br>A **Data Availability Statement**, detailing where the data can be accessed, is required at first submission. Insert your Data Availability Statement in the box below.<br><br>Please see the data reporting section of our submission guidelines for instructions on what you need to include in your Data Availability Statement.<br><br>This statement will be typeset if the manuscript is accepted for publication.<br><br>*PLOS allows rare exemptions to address legal and ethical concerns. If you have legal or ethical restrictions, please use the box below to detail these in full sentences for the Journal team to consider.*	

Manuscript

Click here to access/download;Manuscript;Acceptability low vaginal self-sampling in colposcopy.docx

Full title: Patient preference and acceptability of self-sampling for cervical screening in colposcopy clinic attenders: a cross-sectional semi-structured survey

Short title: Survey of self-sampling acceptability for cervical screening in colposcopy clinic

Sophie Webb[1,2*], Nafeesa Mat Ali[1], Amy Sawyer[2], David J Clark[1], Megan A Brown[1], Yolanda Augustin[1,2], Yin Ling Woo[3,4], Su Pei Khoo[3,4], Sally Hargreaves[1], Henry M Staines[1], Sanjeev Krishna[1,2,3,5,6], Kevin Hayes[1,2,7*]

1. Clinical Academic Group in Institute for Infection & Immunity, St George's University of London, London, UK
2. St George's University Hospitals NHS Foundation Trust, UK
3. Faculty of Medicine, University of Malaya, Kuala Lumpur 59100, Malaysia
4. ROSE Foundation, Kuala Lumpur 50603, Malaysia
5. Institut für Tropenmedizin, Universitätsklinikum Tübingen, Tübingen, Germany
6. Centre de Recherches Médicales de Lambaréné, Gabon
7. Institute for Medical and Biomedical Education, St George's University of London, London, UK\

***Corresponding authors:**

m2109631@sgul.ac.uk (SW)

khayes@sgul.ac.uk (KH)

Abstract

Low vaginal self-sampling has been pioneered as an important development to improve uptake of cervical screening globally. Limited research is available in specific patient groups in the UK exploring views around self-sampling to detect high-risk human papillomavirus (hrHPV) DNA. Therefore, we explored patient views to support development and evaluation of a novel point-of-care self-sampling cervical cancer screening device, by undertaking a cross-sectional semi-structured questionnaire survey to explore preferences, acceptability, barriers and facilitators around self-sampling.

Patients attending a colposcopy clinic (hrHPV negative and positive patients), 25-64 years old, were invited to participate after their appointment, having carried out a low vaginal self-sample. Participants self-completed an anonymous 12-point questionnaire. Quantitative data were analysed in MS Excel and Graphpad Prism whilst qualitative data were analysed using Nvivo.

We recruited 274 patients with a questionnaire response rate of 76%. Acceptability of self-sampling for screening was high (95%, n=187/197; Cronbachs-α =0.778). Participants did not report a strong single preference for future screening method, with 37% (n=74/198) preferring self-sampling, 19% (n=37/198) preferring healthcare professional-collected vaginal swab; 9% (n=17/198) a cervical sample with healthcare professional speculum examination, and 35% (n=70/198) had no preference. Key motivators for self-sampling were: Test simplicity (90%, n=170/190), speed (81%, n=153/190) and less pain (65%, n=123/190). Barriers included lack of confidence taking the sample (53%, n=10/19), resulting in preference for a healthcare professional to take the sample (47%, n=9/19).

Whilst self-sampling showed high acceptability, the lack of strong preference for screening method may reflect that respondents attending colposcopy are already engaged with cervical screening and have a differing perception of cervical cancer risk. This group appear less likely to 'switch' to hrHPV self-sampling, and it may be better targeted within primary and community care settings, focusing on

under-screened populations. Any shift in this paradigm in the UK requires comprehensive education and support for patients and providers.

Introduction

Cervical cancer is the fourth most common cancer in women globally [1]. The majority (99.8%) of cases are caused by persistent high-risk human papillomavirus (hrHPV) infection and are preventable through a combination of HPV vaccination and screening for hrHPV. The World Health Organization (WHO) has called on global stakeholders to work towards cervical cancer elimination worldwide by ensuring 90% of female adolescents are vaccinated, 70% of eligible women are screened with a high-performance HPV test at least twice in a lifetime and ensuring 90% of women who are screened positive receive appropriate follow-up and treatment by 2030 [2]. Limited access to screening, barriers to treatment, and lack of access to HPV vaccination in developing countries [2] are amongst the vital issues that need to be tackled to achieve the WHO's ambition on cervical cancer elimination.

Cervical screening coverage in the United Kingdom continues to decline, with only 68.7% of eligible women screened in 2022-23 (down by 1.2% from the previous year)[3]. This figure was further impacted by the COVID-19 pandemic, which saw screening invitations briefly suspended, and primary care providers given the option to postpone invitations, if needed, for up to 6 months [4]. Along with this, the National Health Service (NHS) – and in particular primary care providers – are facing a crisis, with mounting pressures from long waiting times, staff shortages, and the need to implement catch-up screening. As a result, many women are facing difficulties and delays accessing appointments for cervical screening [5].

In response to the call for solutions to improve access, steps are being taken towards innovative alternative methods of screening, including low vaginal self-sampling for hrHPV. This advance could represent an important landmark in the UK screening programme's evolution, having transitioned to hrHPV nucleic acid amplification tests (NAATs) primary screening from liquid-based cytology in 2019. Low vaginal self-sampling for hrHPV DNA testing for cervical cancer screening has already been implemented nationally in a number of countries worldwide including the Netherlands [6], Malaysia

[7] and Australia [8]. Self-sampling approaches have the aim of tackling well documented common barriers to screening such as embarrassment, pain, fear and preference for a female healthcare practitioner [9].

In a meta-analysis from 2018, self-sampling for hrHPV using PCR-based assays was found to be as accurate as healthcare professional acquired samples in detecting cervical intraepithelial neoplasia grade 2 or worse (CIN2+) [10]. There have been concerns about reduced 'real-world' detection of CIN2+, compared to the meta-analysis, particularly in 'switchers' who currently regularly attend healthcare professional-based screening and may 'switch' to self-sampling. These 'switchers' will only be managed according to their hrHPV status, whereas in primary care they would have the additional information of the cytology result from the healthcare professional acquired cervical brush sample. The reasons for this are not yet fully understood, but it is hypothesized that regular screeners may have more recently acquired hrHPV infections and possibly lower viral load on screening. Another concern is a subsequent lack of adherence to the follow-up cytology sample that would require a healthcare professional-taken sample[11].

The United Kingdom Health Security Agency (UKHSA) is considering offering a choice of self-sampling or healthcare professional acquired sampling to all eligible individuals at the point of invitation [12]. It is important therefore, that evidence is gathered from multiple different populations, on the acceptability and preference for self-sampling in the UK. We therefore aimed to seek the views, acceptability and preferences of a group of women attending a colposcopy clinic at a teaching hospital in south west London that serves an ethnically diverse population (59% and 52% non-white British ethnicity, according to 2021 UK Census data, for Merton and Wandsworth London boroughs respectively[13]). Participants would be attendees at both screening and follow up colposcopy and therefore in the 'switchers' group of interest.

Methods

Design

This cross-sectional survey was designed along-side an on-going study to develop and evaluate a novel point-of-care cervical cancer screening device, to specifically look at acceptability of a self-sampling option for hrHPV detection. Participants who consented to participate in the study on attending their colposcopy appointment at St. George's University Hospitals NHS Foundation Trust, London were asked to complete a semi-structured, anonymous, self-completed questionnaire. Participant enrolment took place prospectively between 13th July 2022 and 19th January 2023.

Ethical approval for participation was provided by the Institutional Review Board as part of the "Development of a prototype device for cervical cancer screening" study, sponsored by St George's University of London (Integrated Research Application System project ID: 235626; South West - Cornwall & Plymouth Research Ethics Committee reference: 18/SW/0244.) All participants were adults, and gave written informed consent to be part of the wider study and questionnaire survey.

Participants undertook a low vaginal self-sample using a Copan FLOQSwab 552c regular flocked swab, followed by a healthcare professional acquired low vaginal swab and an endocervical swab, before proceeding with their routine colposcopy appointment. Participants were a mix of new and follow-up patients, and therefore included both hrHPV positive and negative women. All participants were asked to complete the questionnaire after their appointment. In order to maintain anonymity, personal details and demographics were not recorded on the questionnaire. The questionnaire was estimated to take 10 minutes to self-complete, it was semi-structured and can be reviewed in full in S1 Text appendix. The data was entered into RedCap for ease of analysis and quantitative data were analysed in MS Excel and Graphpad Prism and checked by two members of the study group, whilst qualitative data were analysed with thematic analysis using Nvivo. For quantitative data, an internal reliability analysis was performed for consistency using a Cronbach-α coefficient.

Participants

All attendees at the colposcopy clinic who were eligible at screening and consented to participate in the self-sampling study, between 25-64 years of age, were approached. Exclusion criteria were as follows: a history of cervical cancer; hysterectomy; pregnancy or post-partum less than 4 months; menstruation on the day of clinic; or use of douche/contraceptive cream or NuvaRing® (etonogestrel/ethinyl estradiol vaginal ring)/HRT cream/vaginal pessary for prolapse/Replens (non-hormonal vaginal moisturiser)/thrush cream; severe illness warranting urgent care. On arrival at the clinic, patients were invited to participate in the study, given a patient information sheet (PIS) and allocated time to read and understand the PIS. A member of the study team then approached patients to ask if they had any further questions and if they were willing to participate, written informed consent was taken. Participation in the study had no impact on their clinical care, and they could opt out of the study at any time until they had handed in the questionnaire to the study team.

Validation

The questionnaire used in this study was adapted from a previously validated questionnaire [14,15] that had been used widely in Malaysia[7] to assess acceptability of low vaginal self-sampling for HPV testing in their local population [16,17]. To ensure the questionnaire was suited to a UK clinical setting, we sought input from the public and patient involvement and engagement (PPIE) group at St George's University of London and consulted a group of healthcare professionals including clinical nurse specialists and gynaecology doctors. In response to feedback from members of the public with regards to patient understanding, we re-worded some of the questionnaire items for clarity.

Measures

The primary outcome was to assess acceptability of low vaginal self-sampling and future screening preference. Secondary outcomes were to assess the participant's experience of low vaginal self-

sampling and the reasons for intention to utilise or decline options for low vaginal self-sampling in the future.

The questionnaire consisted of 12 questions (S1 Text), 7 of which used a 5-point Likert scale, one of which was to assess ease of understanding of the patient information sheet for the concurrent novel point-of-care device cervical cancer screening study. The other six questions were aimed indices of the patient experience of taking a low vaginal self-sample, with a score of 1 being the most negative response, and 5 the most positive. A score of 4 or more was considered as a positive response, implying acceptability of self-sampling. Participants were asked about their most preferred method of cervical screening, and if they would be willing to perform a self-sample again as part of future screening and follow up.

There was also a question on feedback for the participant information sheet for the cervical cancer screening study within which the questionnaire was embedded, and a final question with an open text box for any other feedback that the respondents felt to be relevant.

Results

A total of 274 women were initially recruited for the hrHPV POC validation study within which this questionnaire was embedded, of which two withdrew prior to collecting samples and were not therefore invited to complete the questionnaire (Figure 1). The final number of women who completed the questionnaire was 207, a response rate of 76% (207/272). All responses were included in analysis, including those with incompletely filled questionnaires.

Figure 1. Consort diagram for questionnaire response rate

Future screening preference (primary outcome)

Two hundred and seven questionnaires were returned, of which 197 participants (95%) provided a response to the question "Would you be willing to do the HPV self-sampling test again", with the majority (95%, n=187) reporting that they were willing to repeat low vaginal self-sampling in the future.

Having taken their own self-swab for the cervical cancer screening study, women selected their most preferred screening method from 4 choices: Self low vaginal swab, healthcare professional -collected low vaginal swab, 'Pap smear' with healthcare professional performing speculum, or no preference. A total of 198 women responded to this question, with 74 (37%) preferring low vaginal self-sampling, 70 (35%) reporting no preference, 37 (19%) preferring a healthcare professional-collected low vaginal swab and 17 (9%) preferring the traditional speculum examination healthcare professional-collected sample (Table 1).

Table 1. Preferred method of cervical screening, after having attempted a low vaginal self-sample

Which method do you prefer the MOST for cervical screening?	n (total = 198)	%
Self-sampling vaginal swab for HPV testing	74	37%
Healthcare professional - collected vaginal swab for HPV testing	37	19%
Pap smear - healthcare professional conducting a speculum examination	17	9%
No preference	70	35%

Reasons for screening preference

A multiple-choice question ('select all that apply') was asked after the preferred choice of screening, to ascertain motivators and barriers. The main reasons that motivated participants to perform low vaginal self-sampling again in the future were test simplicity (n= 170/190; 90%), speed (n= 153/190; 81%) and that the test was not painful (n= 123/190 ;65%); whilst the main barriers were lack of confidence in taking the sample correctly (n= 10/19 ;53%) and therefore preferring a clinician to take the sample (n= 9/19 ;47%) (Table 2).

Table 2. Motivators and barriers to screening preference

Motivators to hrHPV self-sampling	n	% of respondents (n=190)
Simple	170	90%
Quick	153	81%
Not painful	123	65%
Confident taking accurately	80	42%
More comfortable	63	33%
Less embarrassed	58	31%
**Barriers to hrHPV self-sampling**	**n**	**% of respondents (n=19)**
Not confident taking accurately	10	53%
Prefer clinician to take	9	47%
Not easy	1	5%
Afraid will hurt self	1	5%
Not comfortable	1	5%
Painful	0	0%

The free text response box was analysed for any other factors highlighted that were not included in the main questionnaire. Whilst these responses were predominantly messages of encouragement and praise for the research itself, a small number of additional factors were highlighted; need for more information on accuracy of the result from a self-sample (n= 7/196 [3%]), and access to screening appointments for healthcare professional taken samples was also noted as a barrier (n= 2/196; 1%).

Patient acceptability of self-sampling

The first 6 questions of the questionnaire looked at the various elements of women's experience in taking a low vaginal self-sample, and all six questions were answered by 100% of respondents. An internal reliability analysis was performed which showed good consistency, with a Cronbach-α coefficient of 0.778. Table 3 shows the 1-5 Likert-scale rating responses, and Figure 2 shows a visual representation of these data.

Table 3. Likert-scale responses of patient experience of self-sampling

	Overall experience	Ease	Convenience	Embarrassment	Discomfort /pain	Confidence
**V Positive (5)**	136	163	182	172	139	52
**Positive (4)**	57	37	21	27	45	115
**Neutral (3)**	14	6	4	5	12	34
**Negative (2)**	0	1	0	3	11	5
**V Negative (1)**	0	0	0	0	0	1

Figure 2. 100% Stacked bar charts for Likert-scale responses of patient experience of self-sampling

Women reported their overall experience of self-sampling was positive 93% of the time, and none reported a score of 2 or less. Both ease (97%) and convenience (98%) of self-sampling were also highly rated.

Embarrassment, and pain or discomfort, are known to be common barriers to traditional healthcare professional acquired samples [9], and the majority of women reported that self-sampling was not embarrassing (96%) or painful (89%).

Another common concern, confidence in ability to take a self-sample correctly [9], had the widest spread of responses, and the highest number of 'neutral' responses (16%), but 81% still scored their confidence as positive. Overall, most (92%) women scored the six indices of acceptability of self-sampling with positive answers i.e. 4 or more, indicating a high level of acceptability of this method of screening.

Understanding of the Patient Information Sheet

A single 5-point Likert scale question about participant understanding of the cervical cancer screening study was included at the end of the questionnaire, which showed excellent understanding of the information with 93% (182/195) rating it 4 or greater.

Discussion

This study examined the views of a group of patients who are likely to be regular attenders at screening, given their engagement with secondary care colposcopy services. As such, they are likely to be in the group of potential cervical screening 'switchers' [11] that UKHSA might consider when developing policy and deciding whether hrHPV self-sampling should be aimed at non-attenders or all screen-eligible individuals.

Interestingly, whilst participants indicated their willingness to perform a self-sample again (95%), their future screening preference was more indifferent, with just over one third preferring a self-sample, one third a healthcare professional-taken sample (either with or without speculum), and one third no preference. This is comparable to a recent UK study looking at preferences of all screen-eligible women at the point of invitation, which showed that whilst a higher proportion (59%) of women preferred self-sampling, around a third still felt more confident with a healthcare professional acquired sample [18]. The proportion of women who preferred self-sampling was also lower in our study compared to a recent systematic review [19] of acceptability of hrHPV self-sampling, but the review mostly included studies aimed at capturing screening non-attenders, and this key difference may help to explain the response in the cohort examined in this study. The perception of risk in the sampled population of highly engaged colposcopy clinic attenders may be different to non-attenders, or in women who attend screening but then have a negative hrHPV result, and so may have more trust and confidence in results from healthcare professional-taken samples [18].

The results presented here suggest that whilst around a third of regular screeners are likely to switch to self-sampling, and a third have no preference for self-sampling versus healthcare professional collected samples, a third prefer healthcare professional collected samples still, despite self-sampling being highly acceptable. The 'intention-behaviour gap' [20] may lower this proportion of 'switchers' further; and may alleviate fears of reduced detection of hrHPV in this group; whilst importantly allowing both regular screening attenders, and screening non-attenders a new choice for self-sampling

and thus engagement with the cervical screening programme. It is also worth noting that, whilst not specifically investigated in this study, self-collected urine hrHPV testing is also emerging as a new strategy with good concordance to vaginal samples, which may extend the choices for screening even further, being less invasive and potentially easier to collect [21,22].

In our study, self-sampling for hrHPV DNA was again shown to be highly acceptable (92%), in line with existing literature [19,23], and the main reasons that women were motivated to perform self-sampling again were that it is simple (90%), quick (81%) and not painful (65%); similar to factors cited in comparable studies [19,23]. Barriers were lack of confidence in taking the sample correctly (53%) and preferring a clinician to take the sample (47%), but it was also noted that the respondents were keen for further information on the accuracy of the self-sampling test compared to the pap smear and healthcare professional acquired samples; this important information may influence their decision making and is crucial to consider when rolling out any self-sampling programme in the UK.

Further research needs to be undertaken on the acceptability of self-sampling in specific ethnic minority groups in the UK, as 'never attenders' from these communities may be less likely to choose self-sampling than white women [18], and the reasons around this need to be explored. Our research group is undertaking a follow-on in-depth qualitative research study of such barriers: 'Understanding knowledge, beliefs, values and barriers towards cervical cancer screening, self-sampling and HPV vaccination amongst migrant Muslims and stakeholders in south-west London: An in-depth qualitative interview study' (IRAS reference 306176).

Furthermore, with regards to the impact in low-and-middle-income-countries where the proportion of screening non-attenders is significantly higher [2,17], self-sampling may offer a highly acceptable alternative choice, that does not require a healthcare professional, and may therefore better serve hard to reach communities that face barriers to screening access and HPV vaccination.

There are a number of limitations in this analysis: Lack of data collection on demographics of the respondents was a significant limitation and would have provided increased depth of understanding of the contributory factors to women's screening preference, and there has been work on this in a number of previous studies [9,15,16,17,18,23]. These studies note a likely heterogeneity in results depending on demographics including social background, age, number of partners, and menopausal status for example, and there is a notable lack of acceptability data in participants from more deprived, non-English speaking and ethnic minority groups, which we are attempting to address though the in-depth qualitative study noted above.

Overall, this was a relatively small sample size of 207 respondents, with 195 (94%) completing the questionnaire in full. Whilst this represents a good proportion of those recruited to the larger cervical cancer screening study, and the acceptability data showed good internal reliability, larger sample sizes are needed to give a more accurate reflection of the screening-eligible population which is estimated to be around 5 million women and people with a cervix [3]. It is also noted that the participant cohort may be biased as they had attended initial screening and now colposcopic follow-up, as discussed earlier.

In conclusion, this study shows a highly acceptable method of hrHPV screening in the colposcopy clinic attender population but also the need for information about test accuracy to reduce a major barrier to self-sampling. This method has the potential to ease the burden on the NHS Cervical Screening Programme (NHSCSP) two-fold: Firstly, by reducing the number of face-to-face contacts required for cervical screening (and the need for clinician taken samples); and secondly resulting in a reduced cost of screening [243]. Above all, self-sampling could provide more equitable access to screening in under-screened populations. Further research is needed to understand patient screening preferences and interventions to address cervical health literacy across a diverse population. Any shift in the cervical screening paradigm in the UK requires comprehensive education and support for patients, the general public and healthcare providers.

Acknowledgements

We would like to thank the study participants, and the patients and experts involved in the focus group contributing to the design of the questionnaire, specifically Liberty Lean, Janka Briestenska (Colposcopist), Julie Faerber and Uzma Razat. (from the St. George’s Hospital Charity). We are grateful for the contribution of the colposcopy clinic staff who were always accommodating and motivated to allow time for discussion and informed consent. Finally, the advice and knowledge resource sharing by Program ROSE team at University Malaya, Malaysia was invaluable in the design and implementation of this study.

References:

1. Sung H, Ferlay J, Siegel RL, Laversanne M, Soerjomataram I, Jemal A, et al. Global Cancer Statistics 2020: GLOBOCAN Estimates of Incidence and Mortality Worldwide for 36 Cancers in 185 Countries. CA: a cancer journal for clinicians 2021 May;71(3):209-249.
2. World Health Organisation. Global strategy to accelerate the elimination of cervical cancer as a public health problem. 2020. Available at: https://www.who.int/publications/i/item/9789240014107
3. NHS Digital. Cervical Screening Programme, England - 2022-2023. Available at: https://digital.nhs.uk/data-and-information/publications/statistical/cervical-screening-annual/england-2022-2023. Accessed December, 2023.
4. Castanon A, Rebolj M, Pesola F, Sasieni P. Recovery strategies following COVID-19 disruption to cervical cancer screening and their impact on excess diagnoses. British journal of cancer 2021 Apr;124(8):1361-1365.
5. Jo's Cervical Cancer Trust. The growing issue of access to cervical screening across the UK: The problems and how they can be overcome. 2018; Available at: https://www.jostrust.org.uk/sites/default/files/final_accessibility_policy_jun2018.pdf. Accessed 09/02/, 2023.
6. Aitken CA, van Agt HME, Siebers AG, van Kemenade FJ, Niesters HGM, Melchers WJG, et al. Introduction of primary screening using high-risk HPV DNA detection in the Dutch cervical cancer screening programme: A population-based cohort study. BMC Medicine 2019;17(1):228.
7. Program Rose. Available at: https://www.programrose.org/screening. Accessed 02/02/, 2023.
8. National Cervical Screening Program: Guidelines for the management of screen-detected abnormalities, screening in specific populations and investigation of abnormal vaginal bleeding. 2022 Jul. Available at: https://www.cancer.org.au/clinical-guidelines/cervical-cancer-screening/?title=Guidelines:Cervical_cancer/Screening.
9. Waller J, Bartoszek M, Marlow L, Wardle J. Barriers to cervical cancer screening attendance in England: a population-based survey. Journal of medical screening 2009 Dec;16(4):199-204.
10. Arbyn M, Smith SB, Temin S, Sultana F, Castle P. Detecting cervical precancer and reaching underscreened women by using HPV testing on self samples: updated meta-analyses. BMJ (Online) 2018 Dec 5;363:k4823.
11. Rebolj M, Sargent A, Njor SH, Cuschieri K. Widening the offer of human papillomavirus self-sampling to all women eligible for cervical screening: Make haste slowly. International journal of cancer 2022 Nov 23.
12. PHE Screening. Recruitment for cervical screening self-sampling study now underway. Available at: https://phescreening.blog.gov.uk/2021/04/28/cervical-screening-self-sampling-study/. Accessed 12/02/2023.
13. Office for National Statistics (ONS), released 29 November 2022, ONS website, statistical bulletin, Ethnic group, England and Wales: Census 2021. Population by ethnic group, 2021, local authorities in England and Wales. Accessed November 2023.

14. Dzuba IG, Díaz EY, Allen B, Leonard YF, Lazcano Ponce EC, Shah KV, et al. The Acceptability of Self-Collected Samples for HPV Testing vs. the Pap Test as Alternatives in Cervical Cancer Screening. Journal of women's health & gender-based medicine 2002 Apr 1,;11(3):265-275.

15. Waller J, Mccaffery K, Forrest S, Szarewski A, Cadman L, Austin J, et al. Acceptability of unsupervised HPV self-sampling using written instructions. Journal of medical screening 2006 Jan 1,;13(4):208-213.

16. Ma'som M, Bhoo-Pathy N, Nasir NH, Bellinson J, Subramaniam S, Ma Y, et al. Attitudes and factors affecting acceptability of self-administered cervicovaginal sampling for human papillomavirus (HPV) genotyping as an alternative to Pap testing among multiethnic Malaysian women. BMJ Open 2016 Aug 1,;6(8):e011022.

17. Khoo SP, Lim WT, Rajasuriar R, Nasir NH, Gravitt P, Woo YL. The Acceptability and Preference of Vaginal Self-sampling for Human Papillomavirus (HPV) Testing among a Multi-ethnic Asian Female Population. Cancer prevention research (Philadelphia, Pa.) 2021 Jan;14(1):105-112.

18. Drysdale H, Marlow LA, Lim A, Sasieni P, Waller J. Self-sampling for cervical screening offered at the point of invitation: A cross-sectional study of preferences in England. Journal of medical screening 2022 Sep;29(3):194-202.

19. Nelson EJ, Maynard BR, Loux T, Fatla J, Gordon R, Arnold LD. The acceptability of self-sampled screening for HPV DNA: a systematic review and meta-analysis. Sexually transmitted infections 2017 Feb;93(1):56-61.

20. Faries MD. Why We Don't "Just Do It". American Journal of Lifestyle Medicine 2016 Sep 1,;10(5):322-329. Available at: https://journals.sagepub.com/doi/full/10.1177/1559827616638017.

21. Sargent A, Fletcher S, Bray K, Kitchener HC, Crosbie EJ. Cross-sectional study of HPV testing in self-sampled urine and comparison with matched vaginal and cervical samples in women attending colposcopy for the management of abnormal cervical screening. BMJ open 2019 Apr 1,;9(4):e025388

22. Leeman A, del Pino M, Molijn A, Roiguez A, Torne A, Koning M, et al. HPV testing in first-void urine provides sensitivity for CIN2+detection comparable with a smear taken by a clinician or a brush-based self-sample: cross-sectional data from a triage population. BJOG : an international journal of obstetrics and gynaecology 2017 Aug;124(9):1356-1363.

23. Morgan K, Azzani M, Khaing SL, Wong Y, Su TT. Acceptability of Women Self-Sampling versus Clinician-Collected Samples for HPV DNA Testing: A Systematic Review. Journal of lower genital tract disease 2019 Jul;23(3):193-199.

24. Huntington S, Puri Sudhir K, Schneider V, Sargent A, Turner K, Crosbie EJ, et al. Two self-sampling strategies for HPV primary cervical cancer screening compared with clinician-collected sampling: an economic evaluation. BMJ Open 2023 Jun 6,;13(6):e068940.

S1: Questionnaire

Low Vaginal Self-Sampling Acceptability Questionnaire St George's Hospital Foundation NHS Trust

Figure 1

Click here to access/download;Figure;Fig 1.tif

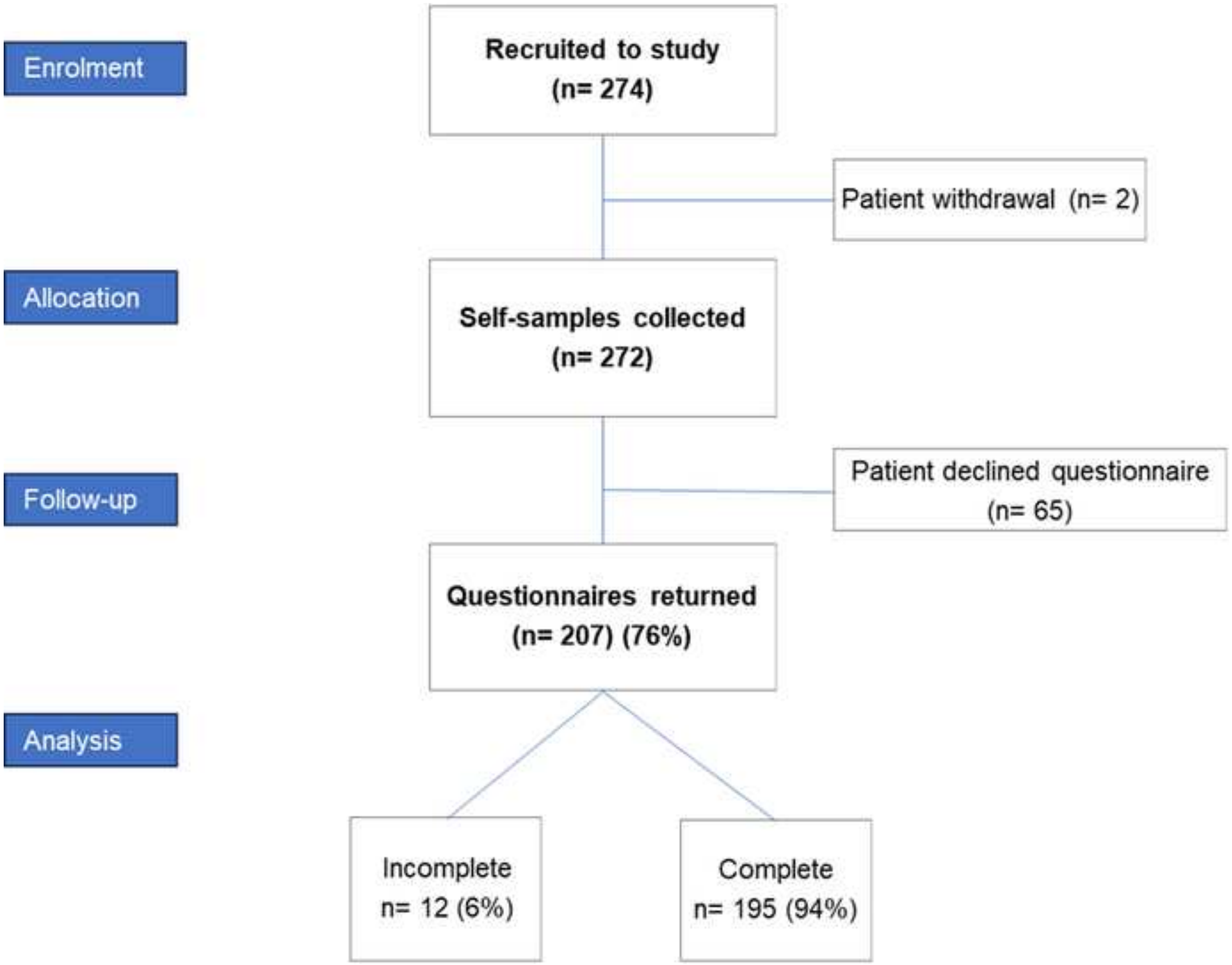

Figure 2

Click here to access/download;Figure;Fig 2.tif

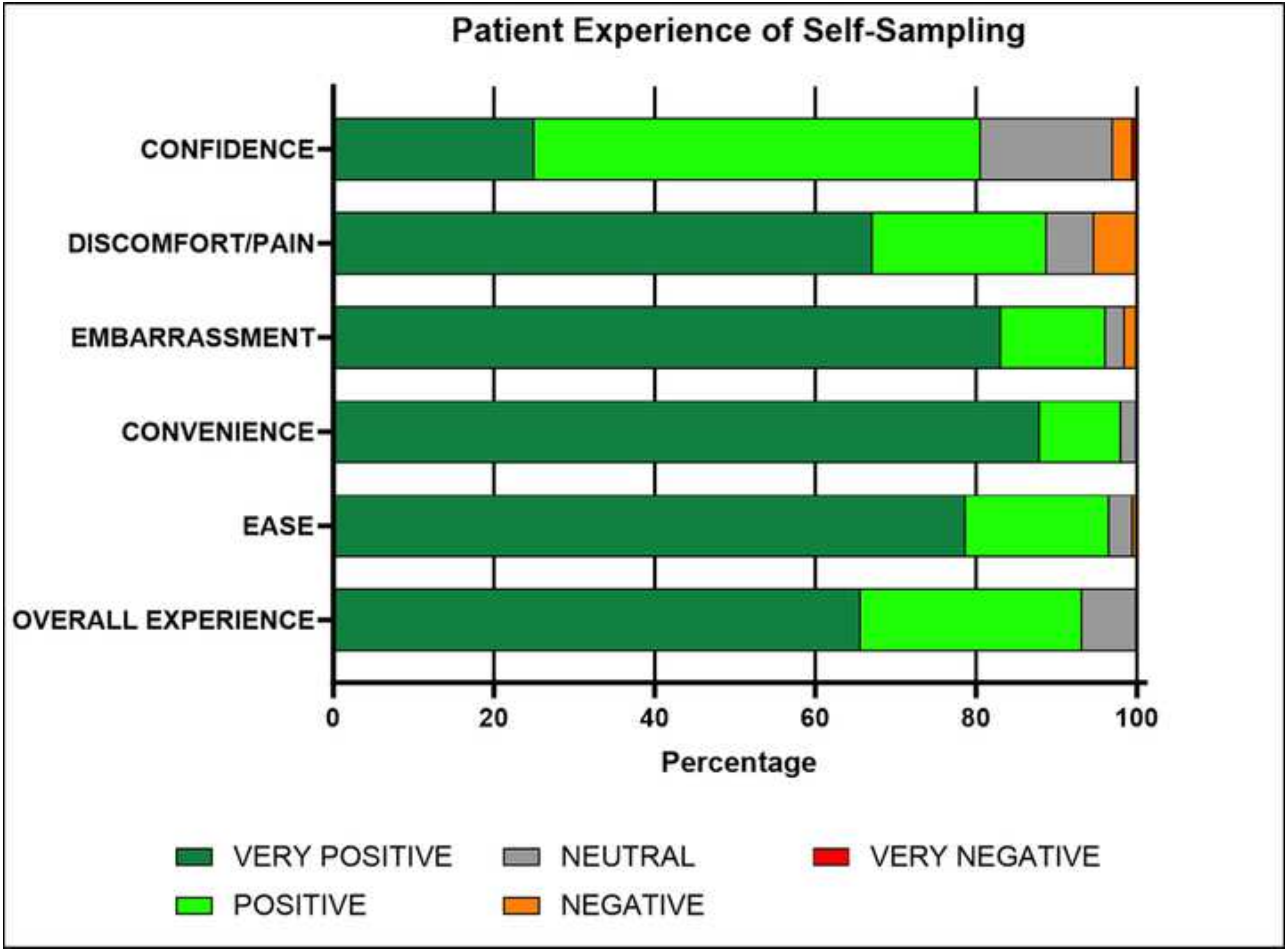

Click here to access/download
Supporting Information
S1 Questionnaire.docx
