# Supplementary material for: Patient preference and acceptability of self-sampling for cervical screening in colposcopy clinic attenders: A cross-sectional semi-structured survey
Source: PLOS Glob Public Health. 2024 May 23;4(5):e0003186. doi: 10.1371/journal.pgph.0003186 (PMC11115198; doi:10.1371/journal.pgph.0003186)
Supplement: Attachment — Submitted filename: Response to Reviewers.docx [file pgph.0003186.s004.docx]

**Journal Requirements:**

1. Please include a complete copy of PLOS’ questionnaire on inclusivity in global research in your revised manuscript. Our policy for research in this area aims to improve transparency in the reporting of research performed outside of researchers’ own country or community. The policy applies to researchers who have travelled to a different country to conduct research, research with Indigenous populations or their lands, and research on cultural artefacts. The questionnaire can also be requested at the journal’s discretion for any other submissions, even if these conditions are not met. Please find more information on the policy and a link to download a blank copy of the questionnaire here: https://journals.plos.org/globalpublichealth/s/best-practices-in-research-reporting. Please upload a completed version of your questionnaire as Supporting Information when you resubmit your manuscript.

The completed copy of the PLOS’ questionnaire on inclusivity in global research has been added to the manuscript.

2. We do not publish any copyright or trademark symbols that usually accompany proprietary names, eg ©, ®, ™ (e.g. next to drug or reagent names). Please remove all instances of trademark/copyright symbols throughout the text, including ® on page 7.

Copyright/trademark symbols have been removed from the manuscript.

**Response to the reviewers:**

We thank the Reviewers for their time and considered comments and have responded to each below:

**Reviewer #1:** Patient preference and acceptability of self-sampling for cervical screening in colposcopy clinic attenders: a cross-sectional semi-structured survey

Line 36-40: The authors should first start the paragraph by explaining the variations in options of self-sampling available to the participants before describing the proportion who accepted what type of sampling. This will allow clarity in the study and make it easier to understand.

We thank the Reviewer for their suggestion and this information has been added to the relevant section, which now reads:

“We recruited 274 patients with a questionnaire response rate of 76%. Acceptability of self-sampling for screening was high (95%, n=187/197; Cronbachs-α =0.778). Participants were asked their choice of future screening method: a) Low vaginal self-sampling, b) a healthcare professional collected vaginal swab, c) a cervical brush sample with healthcare professional speculum examination, or d) no preference.”

“”

Line 44: What does the author mean by “the lack of strong preference for screening method…” do you mean those who did not have any preference?

We apologise for the confusion here. We mean to say that opinions were divided amongst the different options for screening method, with no one option standing out as a clear preferred choice over the others.

This point has been clarified with the following text: “Preferences were as follows: a) 37% (n=74/198), b) 19% (n=37/198); c) 9% (n=17/198), and d) 35% (n=70/198), showing no single option as a strong preference.”

Line 61: This paragraph reviewing the decline in the screening rate in the recent years should also discuss aside COVID-19 what are the other possible factors that could lead to the decline in the screening rate, this can further buttress the need for the self-sampling technique and justify the need for this study. This was explained briefly in line 75 to 77 however, it was not detailed.

We thank the Reviewer for their suggestion and this information has been added to the relevant section, which now reads:

“Low awareness and understanding of screening are also factors in the declining screening rates, with 1 in 5 women in the UK unaware that cervical screening does not detect ovarian cancer [6] [7]. Inequalities in access to screening remains a challenge, with 63% of physically disabled women reporting being unable to attend screening [8], and 80% of women in full-time employment unable to find a convenient appointment [7]. Furthermore, women in areas with higher levels of deprivation [9], and survivors of sexual violence [10] are also less likely to attend screening. A 2023 report by Jo’s Cervical Cancer Trust, which surveyed 848 individuals working within cervical cancer and prevention in the UK [7] highlights these challenges, and found that the main barriers to screening were: Anxieties among the eligible population (66%), workforce pressures in primary care (63%), low levels of understanding among the public (62%), inaccessibility for some of the eligible population (48%) and lack of digitalisation (32%).”

Method section, Measures; the available choices of sampling for the participants should be described when explaining the Questionnaire.

We thank the Reviewer for their suggestion and this point has been clarified with the following text:

“Participants were then asked about their most preferred method of cervical screening (from the following options: a) low vaginal self-sampling, b) a healthcare professional collected vaginal swab, c) a cervical brush sample or ‘pap smear’ with healthcare professional speculum examination, or d) no preference.), and if they would be willing to perform a low vaginal self-sample again as part of future screening and follow up.”

I would also suggest that a future recommendation should include experimental studies of training woman on self-sampling and accessing their level of confidence before and after the training. Evidence of this is in the result section, 53% didn’t have confidence and also the 43% Preference for clinician to take needs to still be explored, why the increased number of preference for a clinician.

We thank the Reviewer for their suggestion and this information has been added to the relevant section, which now reads:

“This is in line with previous findings by Jo’s Cervical Cancer Trust [7] and bolsters the need for national awareness campaigns, increased education on screening and HPV, and indicates the need for further research on methods of teaching women to self-sample and the effect of this on confidence in screening method and choice of future screening method.”

This study is a very relevant one and will bring about a shift in the healthcare sector, which is relevant for cervical screening, but I believe the decline in the self-sampling preference will have a lot to do with self-confidence of performing the procedure.

Great work!

We thank the Reviewer for their kind comments on our paper.

Reviewer #2: I have three main comments.

Line 94: This needs to be clarified. Are there two locations with 59% and 52% non-white British ethnicity?

We apologise for the confusion here and this point has been clarified with the following text:

“We therefore aimed to seek the views, acceptability and preferences of a group of women attending a colposcopy clinic at a teaching hospital in south west London that serves an ethnically diverse population (London boroughs of Merton - 59% non-white British, and Wandsworth - 52% non-white British ethnicity, according to 2021 UK Census data [18]).”

Line 170: This is an important finding, especially given the racial diversity of the sample and the relatively high levels of racism in healthcare in Britain.

We thank the Reviewer for their kind comments on this finding.

Line 262: I wish a deeper analysis on race in health in Britain could have been examined here, even if 2-3 sentences. There is a wealth of literature on this.

We thank the Reviewer for their suggestion and have added the following text to the relevant section, which now reads:

“Further research also needs to be undertaken on the acceptability of self-sampling in specific ethnic minority groups in the UK, as cervical screening ‘never attenders’ from these communities may be less likely to choose self-sampling than white women [23], and the reasons around this inequity need to be explored. This is particularly important in the context of declining UK screening numbers, as women from ethnic minority groups in the UK have been associated with overall lower screening attendance [29] [30], with fear of pain, embarrassment and need for a female healthcare professional noted as barriers, to which hrHPV self-sampling may provide an alternative option to bridge these gaps.”

Reviewer #3: This is an important study on Patient preference and acceptability of self-sampling for cervical screening in colposcopy clinic attenders: a cross-sectional semi-structured survey

The study aims to seek views of the end users on a new point of care.

The manuscript is well written and has coherence

The methods and results are well presented

The conclusion is in keeping with the aim of the study

The authors may clarify in the discussion

How was your study different from the one from Malaysia from which your methods were adopted

We thank the Reviewer for their query.

This information has been added to the relevant section, which now reads:

“In response to feedback from members of the public with regards to patient understanding, we re-worded some of the questionnaire items for clarity, to make it more relevant to the UK population, and also added a free text section at the end to allow for additional comments that participants felt were pertinent. Unlike the Malaysian studies we did not take any pre-sampling demographics and the questionnaire was only performed following the self-sampling, as opposed to before and after, and our study population were specifically attending a colposcopy clinic for themselves, as opposed to a more opportunistic approach in the Malaysian study.”

What are the strength of this study?

We thank the Reviewer for their suggestion and this information has been added to the Discussion, which now reads:

“Strengths of this study however, are that this represents a good proportion of those recruited to the larger cervical cancer screening study, and the acceptability data showed good internal reliability, but larger sample sizes are needed to give a more accurate reflection of the screening-eligible population which is estimated to be around 5 million women and people with a cervix [3]. It is also noted that the participant cohort may be biased as they had attended initial screening and now colposcopic follow-up, as discussed earlier, though this does provide a unique insight into a population from a diverse area of London, who are mostly referred with cervical cell changes on pap smear, and may need increased surveillance.”
